# Promotion of Glioblastoma Cell Motility by Enhancer of Zeste Homolog 2 (EZH2) Is Mediated by AXL Receptor Kinase

**DOI:** 10.1371/journal.pone.0047663

**Published:** 2012-10-15

**Authors:** Martina Ott, Ulrike M. Litzenburger, Felix Sahm, Katharina J. Rauschenbach, Ruxandra Tudoran, Christian Hartmann, Victor E. Marquez, Andreas von Deimling, Wolfgang Wick, Michael Platten

**Affiliations:** 1 Department of Neurooncology, University Hospital Heidelberg, Heidelberg, Germany; 2 Institute for Neuropathology, University Hospital Heidelberg, Heidelberg, Germany; 3 Helmholtz Group Experimental Neuroimmunology and Clinical Cooperation Unit, German Cancer Research Center, Heidelberg, Germany; 4 Department of Neurooncology, German Cancer Research Center, Heidelberg, Germany; 5 Department of Neuropathology, German Cancer Research Center, Heidelberg, Germany; 6 Department of Neuropathology, Institute for Pathology, Hannover Medical School, Hannover, Germany; 7 Chemical Biology Laboratory, National Cancer Institute, Frederick, Maryland, United States of America; Ospedale Pediatrico Bambino Gesu', Italy

## Abstract

Enhancer of zeste homolog 2 (EZH2) is the catalytic subunit of the Polycomb-repressive complex 2 (PRC2) that epigenetically silences gene transcription through histone H3 lysine trimethylation (H3K27me3). EZH2 has been implicated in stem cell maintenance and is overexpressed in hematological and solid malignancie`s including malignant glioma. EZH2 is thought to promote tumor progression by silencing tumor suppressor genes. Hence pharmacological disruption of the PRC2 is an attractive therapeutic strategy for cancer treatment. Here we show that EZH2 is expressed in human glioma and correlates with malignancy. Silencing of EZH2 reduced glioma cell proliferation and invasiveness. While we did not observe induction of cell cycle-associated tumor suppressor genes by silencing or pharmacological inhibition of EZH2, microarray analyses demonstrated a strong transcriptional reduction of the AXL receptor kinase. Neither histone nor DNA methylation appeared to be involved in the positive regulation of AXL by EZH2. Silencing AXL mimicked the antiinvasive effects of EZH2 knockdown. Finally, AXL expression is found in human gliomas with high EZH2 expression. Collectively these data suggest that EZH2 drives glioma invasiveness via transcriptional control of AXL independent of histone or DNA methylation.

## Introduction

Enhancer of zeste homolog 2 (EZH2) is the catalytic subunit of the Polycomb-repressive complex 2 (PRC2) and centrally involved in epigenetically regulating gene transcription programs during development and cellular differentiation [1]. EZH2 acts mainly through trimethylation of histone H3 lysine27 (H3K27me3), which is associated with transcriptional repression. Furthermore this modification facilitates the recruitment of a second Polycomb repressor complex (PRC1), of DNA methyltransferases (DNMT) and of histone deacetylases (HDACs), resulting in chromatin compaction [2]. In the hematopoietic system EZH2 represents a crucial checkpoint controlling self renewal, differentiation and aging [3]. With the emerging concept of tumor stem cells it has subsequently become clear that EZH2 similarly controls expansion and differentiation of tumor initiating cells [4] and contributes to the development and progression of cancer [2], [5]. Inactivating mutations in the *EZH2* gene in myelodysplastic syndromes are frequent and point to a general function of EZH2 as a tumor suppressor [6], [7]. In malignant gliomas EZH2 is upregulated [8] and maintains stemness of tumor cells by inhibiting their differentiation [9], [10]. Consequently, inhibition of *EZH2* by short hairpin RNA (shRNA)-mediated knockdown or 3-Deazaneoplanocin A (DZNep) suppresses growth in glioma animal models [10], [11]. These therapeutic approaches have indicated that EZH2 controls diverse phenotypic features of cancer including proliferation, invasiveness, metastasis and resistance to cell death [12], [13], [14], [15]. While global transcriptional profiling studies have been undertaken to identify the target genes involved in the EZH2-mediated promotion of cancer [5], the multitude of functionally relevant genes identified in various types of tumors indicate that the molecular and functional consequences of EZH2 in cancer heavily depends on the cellular, developmental context and even extends to non-transformed host tissue [16], [17]. While most studies have identified cancer-suppressing target–mostly cell cycle-associated–genes that are repressed by EZH2 through epigenetic silencing [18], few studies have shown tumor-promoting genes that are positively regulated by EZH2 such as c-myc in glioblastoma [10]. Here we identify a novel target gene in glioblastoma that is positively regulated by EZH2 and mediates invasiveness driven by EZH2.

## Materials and Methods

### Cells and reagents

The human malignant glioma cell lines LN18 and A172 were kindly provided by N. De Tribolet (Lausanne, Switzerland) and the human malignant glioma cell line U87MG was a kind gift of A. Abdollahi (Heidelberg, Germany) [18], [19]. The glioma cell lines were cultured in Dulbecco's modified Eagle's medium (DMEM) containing 10% fetal bovine serum (FBS), penicillin (100 U/ml) and streptomycin (100 mg/ml) (all PAA, Laboratories, Pasching, Austria). The glioma initiating cells (GIC) S24 and T269 were established from freshly resected tumors and used during the first passages [20]. The GIC culture methods were modified from the study of Svendsen et al [21]. GIC were cultured in DMEM containing B27 supplement (both Invitrogen, Darmstadt, Germany), leukemia inhibitory factor (LIFF, Invitrogen), heparin (Sigma-Aldrich, Taufkirchen, Germany), basic fibroblast growth factor (bFGF, Invitrogen) and epidermal growth factor (EGF, R&D Systems, Wiesbaden, Germany). Human astrocytes were obtained from ScienCell (Carlsbad, CA, USA) and cultured in astrocyte medium (ScienCell). Human mesenchymal stem cells (MSC) were obtained from bone tissues from total hip replacement surgeries of nine different patients following informed consent. After density gradient centrifugation, MSC isolated by plastic adherence were grown in basal medium for human MSC with 10% stimulatory supplement (CellSystems, St. Katharinen, Germany) [22]. Passages 4–11 were used for experiments. 5-aza-2′-deoxycytidine (5-aza) and Suberoylanilide hydroxamic acid (SAHA) are from Sigma (Sigma-Aldrich) and Trichostatin A (TSA) from Cayman chemicals (Hamburg, Germany). Stock solutions of 5-aza, SAHA and TSA were prepared in dimethyl sulfoxide (DMSO), while DZNep [23] was dissolved in water. All cells were routinely tested for contamination by the Multiplex cell Contamination Test (McCT) [19], [24].

### Matrigel invasion assay

Invasion assays were conducted as previously described [19]. To analyze the invasion of the glioma cells matrigel-coated Boyden chambers of 8 µm pore size (BD Biosciences, Heidelberg, Germany) were used. 6×10^4^ glioma cells were seeded in 200 µl culture medium into the upper part of each chamber, and conditioned medium (0.5 ml) derived from NIH-3T3 fibroblasts was added to the lower chamber as a chemoattractant. Cells were incubated at 37°C and allowed to transmigrate through the membrane. After 24 h the non-migrated cells in the upper chamber were removed using a cotton swab and the migrated cells on the lower side of the membrane were fixed with methanol and stained with crystal violet (Carl Roth, Karlsruhe, Germany). The migrated cells were quantified by counting the number of cells that had migrated across the membrane in five independent microscopic high-power fields and expressed as percentage of invasiveness relative to control using a microgrid. Experiments were performed in triplicates.

### Quantitative (q) RT-PCR

QRT-PCR analyses were performed as previously described [25]. Briefly, total RNA was prepared using the Qiagen RNAeasy RNA isolation kit (Hilden, Germany). 1 µg total RNA was used to synthesize cDNA with the Applied Biosystems reverse-transcription-Kit (Foster City, CA, USA) according to manufacturer's instructions. QRT-PCR was performed in an ABI 7000 thermal cycler according to standard protocols with SYBR Green PCR Mastermix (Applied Biosystems, Carlsbad, USA). Glyceraldehyde-3-phosphate dehydrogenase (*GAPDH*) was used as housekeeping gene. All primers were separated by one intron on the genomic DNA to exclude amplification of genomic DNA. PCR reactions were checked by omission of templates, by melting curves and by agarose gel electrophoresis. Standard curves were generated for each gene. Relative quantification of gene expression was determined by comparison of threshold values. All samples were analyzed in duplicate in two different dilutions. All results were normalized to *GAPDH*. The primer sequences are shown in Table S1.

### Small interfering RNA (siRNA) experiments

For the transient knockdown of *AXL* SMART-pool siRNA by Dharmacon RNA Technologies (Lafayette, CO, USA) was used. For the transient knockdown of *EZH2* siRNA was generated by Dharmacon against the EZH2 mRNA target sequence: 5′-AAGACTCTGAATGCAGTTGCT-3′ [14]. ON-TARGETplus siCONTROL Non-targeting Pool (D-001810-10-05, Dharmacon) and a transfection without siRNA served as negative controls. The transfections were performed with lipofectamine RNAiMAX from Invitrogen and the knockdown efficiency was verified by qRT-PCR and Western Blot. The *AXL* target sequences were as follows:

5′-GAAGGAGACCCGUUAUGGA-3′; 5′-GGUACCGGCUGGCGUAUCA-3′;

5′-GACGAAAUCCUCUAUGUCA-3′; 5′-ACAGCGAGAUUUAUGACUA-3′

### Western Blot analysis

For EZH2 detection, cells were lyzed in ice cold tris (hydroxymethyl) aminomethane hydrochloride (TRIS-HCl, 50 mM, pH 8,0; Carl Roth) containing 150 mM NaCl (J.T. Baker, Deventer, Netherlands), 1% Nondiet P-40 (Genaxxon Bioscience, Ulm, Germany), 10 mM Ethylenediaminetetraacetic acid (EDTA) (GerbuBiotechnik, Gaiberg, Germany), 200 mM dithiothreitol (Carl Roth), 100 µM phenylmethylsulphonyl fluoride (PMSF) and complete EDTA-free (1∶50, Roche, Mannheim Germany) for 20 min and then centrifuged for 10 min (4°C, 13 000 rpm). For H3K27me3 Western Blot, cells were lysed in ice-cold RIPA-Buffer (50 mM TRIS-HCl pH 7.4, 150 mM NaCl, 5 mM EDTA, 1% Triton-X 100, 1% Sodium deoxycholate (both AppliChem GmbH, Darmstadt. Germany), 100 µM PMSF and complete EDTA-Free (1∶50) for 20 min, sonicated and then centrifuged for 10 min (4°C, 13 000 rpm). Protein concentrations were determined with the Bio-Rad protein assay (Bio-Rad, Hercules, CA, USA) at 595 nm. The proteins were separated by 10% (anti-EZH2 Western Blots) or 15% (anti-H3K27me3 Western Blot) sodium dodecyl sulfate-polyacrylamide gels. Proteins were transferred onto a nitrocellulose membrane (Whatman, Dassel, Germany) at 1.5 mA/cm^2^ for 1 h 20 min (EZH2 Western Blot) or onto polyvinylidene fluoride membrane (PVDF, Millipore, Temecula, CA, USA) for 45 min (H3K27me3 Western Blot). After 1 h blocking in tris-based saline (TBS) supplemented with 0.1% Tween 20 (Sigma-Aldrich) and 5% milk powder (Carl Roth), the membranes were incubated with monoclonal mouse anti-EZH2 (1∶1000, Cell Signaling Technology, Beverly, USA), polyclonal rabbit anti- trimethylation of histone H3 lysine27 (H3K27me3) (1∶2000, Millipore) or with monoclonal mouse anti-α-tubulin (1∶5000, Sigma–Aldrich) overnight at 4°C. Staining with secondary horseradish peroxidase conjugated anti-mouse or anti-rabbit antibody at dilutions of 1∶5000 (both GE Healthcare, Buckinghamshire, UK) was followed by immunodetection with ECL Plus reagent (GE Healthcare).

### Immunohistochemistry

Formalin-fixed paraffin-embedded tissue of human diffuse astrocytomas (WHO grade II, n = 4 for AXL staining, all Isocitrate dehydrogenase 1 (IDH1) mutated (IDH1R132H as evidenced by immunohistochemistry [26]); n = 10 for EZH2 staining, 7 with identified IDH1 mutation, remaining unknown), anaplastic astrocytomas (WHO grade III, n = 5 for AXL staining, all IDH1 mutated; n = 15 for EZH2 staining, 8 IDH1 mutated, remaining unknown) and glioblastoma (WHO grade IV, n = 12 for AXL and EZH2 staining, one IDH1 mutant and 11 wild type) were provided by the Department of Neuropathology, Institute of Pathology, University Hospital Heidelberg, Germany. Written informed consent was given by patients and the analyses were approved by the local ethics committee approving the biobanking and tissue analyses. Sections cut to 3 µm were processed using a Ventana BenchMark XT® immunostainer (Ventana Medical Systems, Tucson, AZ, USA). Staining procedure included a pretreatment with either cell conditioner 1 (pH 8) for 60 min for EZH2 or cell conditioner 2 (pH 6) for AXL, respectively. Pre-treatment was followed by incubation with either rabbit anti-human EZH2 antibody (1∶100; Invitrogen) or rabbit anti-human AXL (1∶50; Sigma-Aldrich) at 37°C for 32 min. Incubation was followed by Ventana standard signal amplification, UltraWash, counterstaining with one drop of hematoxylin for 4 min and one drop of bluing reagent for 4 min. For visualization, ultraView™Universal DAB Detection Kit (Ventana Medical Systems) was used. For quantitative analysis of staining pattern, the Histo-Score adapted from Bruna et al. was applied [27]. The score ranges from 0 to 300 and is calculated as the percentage of weakly stained cells plus the percentage of moderately stained cells multiplied by two plus the percentage of strongly stained cells multiplied by three.

### Cell cycle analysis

Cells were treated as indicated and incubated with 10 µM bromodeoxyuridine (BrdU, BD Bioscience, Heidelberg, Germany). After 1 h the cells were harvested, fixed in ice-cold methanol and treated with a PBS-based buffer containing 0.1 M hydrochloric acid (VWR) and 0.3% Triton X-100 for 10 min at 4°C, followed by boiling in water to uncover the DNA. Finally, the cells were stained with Alexa-Fluor 647 mouse anti-BrdU antibody (clone 3D4, 1∶50, BD Bioscience) for 30 min at room temperature and 20 µg/ml DAPI (Sigma-Aldrich) was added 1 min before analysis by flow cytometry using a BD FACS CANTO II cytometer (BD Biosciences).

### Flow cytometry

To analyze the toxicity of DZNep, annexin-V/DAPI staining was performed and apoptosis was quantified by flow cytometry. After treatment of the cells with the indicated concentrations of DZNep for 120 h cells were collected, washed with PBS and the cell pellets were solved in Annexin-V-FITC and DAPI containing Annexin-binding buffer, incubated for 5 min at room temperature and the resulting fluorescence was measured by flow cytometry using a BD FACS CANTO II cytometer. For quantification of the nestin expression in S24 GIC, cells were treated with 5 µM DZNep, after 120 h trypsinized and washed with PBS containing 3% FBS and 2 mM EDTA, followed by fixation and permeabilization using the BD Cytofix/Cytoperm solutions (BD Bioscience), according to the manufacturer's protocol. Finally cells were stained with Alexa Fluor 647 mouse anti-nestin (1∶30, BD Bioscience) or Alexa Fluor 647 mouse IgG k Isotype control (1∶15, BD Bioscience) for 30 min on ice and analyzed by flow cyotmetry. For analysis of AXL cell surface expression cells were harvested after the indicated incubation times by accutase treatment (PAA), washed with PBS containing 3% FBS and 2 mM EDTA, followed by 45 min incubation with polyclonal goat anti-AXL or polyclonal goat IgG (2.5 µg/ 10^6^ cells, R&D Systems) on ice. Finally, cells were stained with the secondary Alexa Fluor 488 donkey anti-goat antibody (1∶200, Invitrogen) for 30 min on ice, and 20 µg/ml DAPI (Sigma-Aldrich) was added 1 min before analysis by flow cytometry using a BD FACS CANTO II cytometer.

### Microarray Analysis

Total RNA from U87MG glioma cells was isolated as described above. The quality of total RNA was checked by gel analysis using the total RNA Nano chip assay on an Agilent 2100 Bioanalyzer (Agilent Technologies GmbH, Berlin, Germany). Only samples with RNA index values greater than 8.5 were selected for expression profiling. Biotin-labeled cRNA samples for hybridization on Illumina Human Sentrix-12 BeadChip arrays (Illumina, Inc.) were prepared according to Illumina's recommended sample labeling procedure based on the modified Eberwine protocol [28]. Microarray scanning was done using an iScan array scanner. Data extraction was done for all beads individually, and outliers are removed when > 2.5 MAD (median absolute deviation). All remaining data points are used for the calculation of the mean average signal for a given probe, and standard deviation for each probe was calculated. The analysis was done using standard algorithms for outlier removal, averaging values, and statistical tests as previously described [19]. Quantile-normalized gene expression measurements were log2 transformed. Multiple probes per gene were averaged. Log fold-change between treated and untreated samples was calculated. For siRNA treatment the ten most regulated genes in terms of absolute log fold change were displayed in a heatmap using complete linkage and Euclidian distance. All analyses were performed with the software R 2. (http://www.R-project.org/).

### Statistical analysis

Data are expressed as mean ± s.e.m. and analysis of significance was performed using the Student's t-test (Microsoft Office Excel). P values < 0.05 were considered significant. Box-plots were generated with SigmaPlot. Correlations were analyzed by Spearman rank correlation (SigmaPlot).

## Results

### EZH2 expression in human malignant glioma

**Figure 1:**
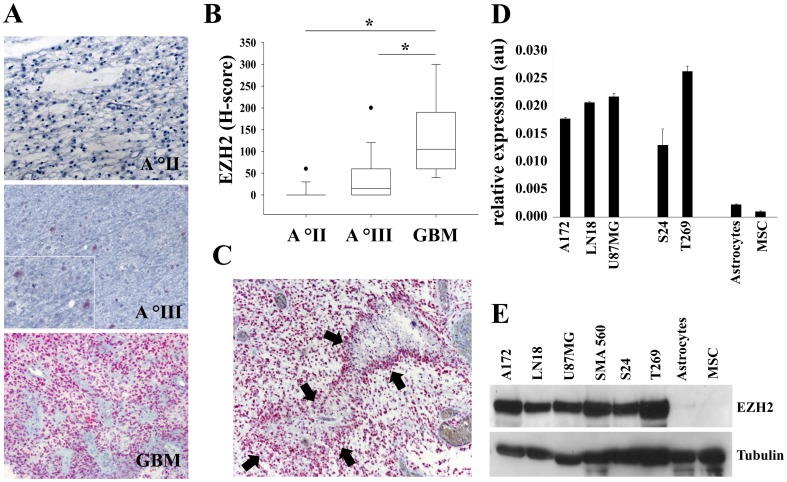
EZH2 expression in human malignant glioma. **A** EZH2 expression (red) in human glioma sections of different WHO grades: astrocytoma grade II (top), astrocytoma grade III (middle), glioblastoma (bottom). Magnification 200x, inset 400x. **B** Box-plot of EZH2 expression in human brain tumors of increasing malignancy (WHO grade II: n = 10, mean = 6, SD = 18.97; WHO grade III: n = 15, mean = 40.67, SD = 56.91; GBM: n = 12, mean = 135, SD = 98.59; r^2^ = 0.385, P = 0.024). **C** EZH2 expression (red) in glioblastoma sections in close proximity to necrotic areas. Arrows indicate the necrotic area. Magnification 100x. **D** *EZH2* mRNA expression in A172, LN18, U87MG human malignant glioma cells, S24, T269 human glioma-initiating cells (GIC), human astrocytes and human mesenchymal stem cells (MSC) measured by qRT-PCR. **E** EZH2 protein expression in A172, LN18, U87MG human malignant glioma cells, S24, T269 human GIC, human astrocytes and human MSC. Tubulin served as loading control. Asterisk indicates * (p<0.05). Error bars indicate s.e.m.

We first analyzed EZH2 expression in glioma by immunohistochemistry. EZH2 showed a strong nuclear staining pattern as opposed to normal brain tissue (Figure 1A). Both, number of positive nuclei and staining intensity positively correlated with the degree of malignancy (Figure 1B; r^2^ = 0.385 P = 0.024). EZH2 expression was stronger in perinecrotic areas as opposed to the invasion front (Figure 1C). Compared with human untransformed astrocytes and mesenchymal stem cells (MSC) *EZH2* mRNA and protein expression is higher in glioma cell lines and glioma initiating cells (GIC) (Figure 1D, E). The used human GIC S24 and T269 form neurospheres under the indicated culture conditions (Figure S1). Collectively, these data indicate that EZH2 is associated with a malignant phenotype in gliomas.

### EZH2 knockdown inhibits proliferation and invasion of human malignant glioma cells

**Figure 2:**
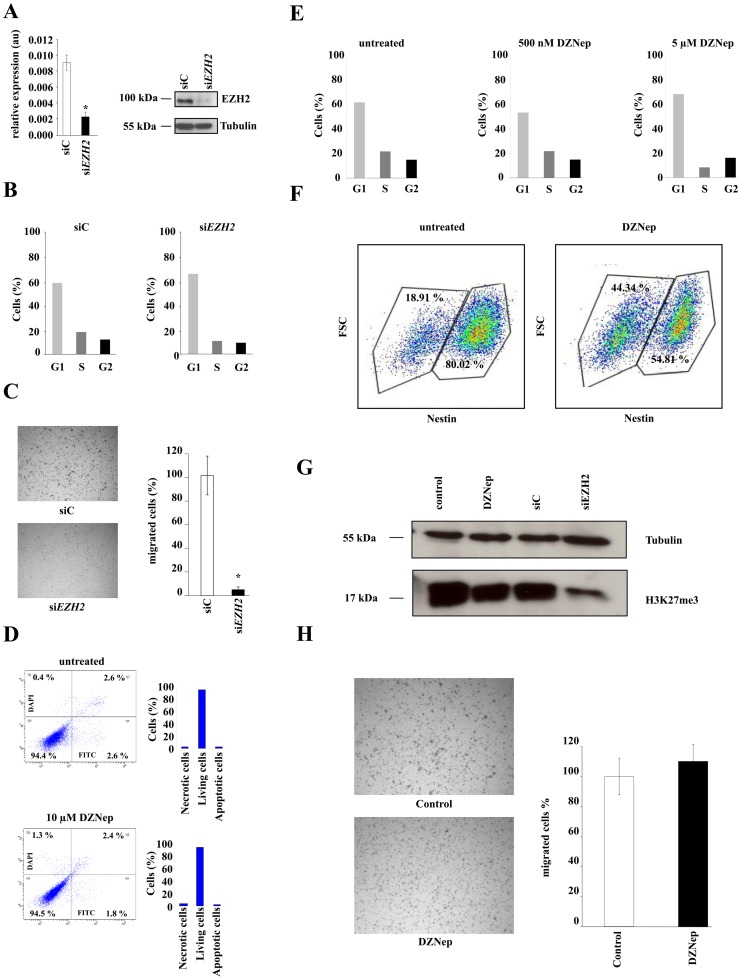
EZH2-knockdown inhibits proliferation and invasion of human malignant glioma cells. **A** *EZH2* transcript expression was decreased 24 h after si*EZH2* treatment (left). Western blot showing EZH2 protein expression, 120 h after knockdown by siRNA (right). Tubulin served as loading control. **B** Cell cycle analysis of U87MG glioma cells 120 h after specific knockdown of *EZH2* (si*EZH2*, right) or scrambled control (siC, left). **C** Invasion of U87MG glioma cells with transient *EZH2* knockdown (lower panel, black bar) through a matrigel-coated boyden chamber in comparison to control (upper panel, white bar). **D** Representative dot plots and corresponding analysis of Annexin-V-FITC/DAPI co-staining of U87MG glioma cells untreated or treated for 120 h with 10 µM DZNep. The lower left quadrants represent the living cells (low Annexin-V-FITC-/DAPI-signal), the lower right quadrants represents early apoptosis (low DAPI- and strong Annexin-V-FITC-signal) and the upper right late apoptotic/necrotic cells (double-stained cells). **E** H3K27me3 methylation was strongly decreased in whole cell lysates of U87MG glioma cells after treatment with 5 µM DZNep or after specific knockdown of *EZH2* for 120 h. Tubulin served as loading control. **F** Cell cycle analysis of U87MG glioma cells untreated or treated for 120 h with 500 nM and 5 µM DZNep. **G** Analysis of nestin expression in S24 glioma-initiating cells untreated (left) or treated with 5 µM DZNep for 120 h (right) by flow cytometry. **H** Matrigel boyden chamber assay of U87MG glioma cells untreated (upper panel, white bar) and treated (lower panel, black bar) with 5 µM DZNep. Asterisk indicates * (p<0.05). Error bars indicate s.e.m.

To characterize the functional significance of EZH2 in glioma cells we used siRNA to knockdown *EZH2* in U87MG glioma cells. The knockdown of *EZH2* was stable for 120 h (Figure 2A) and reduced cell proliferation by causing a G1 phase arrest (Figure 2B). More prominent than the antiproliferative effect, however, was the antiinvasive properties of *EZH2* knockdown in Boyden chamber assays. *EZH2* knockdown in U87MG cells resulted in a reduction of invaded cells by 80% (Figure 2C). To investigate whether the antiinvasive effect of EZH2 knockdown is methylation-dependent, we treated U87MG glioma cells with the histone methylation inhibitor 3-Deazaneoplanocin A (DZNep). DZNep inhibits the S-adenosylhomocysteine hydrolase, a component of the methionine cycle in which the methyl-donor for the methyltransferase reaction is produced. No toxic effects were observed in U87MG cells after DZNep treatment using concentrations, which have been reported to be sufficient for an inhibition of the methyltransferase activity of EZH2 (Figure 2D). Cell proliferation of U87MG cells was suppressed after treatment with 5 µM DZNep to the same extent as after the specific knockdown of *EZH2* (Figure 2E). Of note, in S24 GIC treatment with DZNep led to a downregulation of the stem cell marker nestin (Figure 2F), which is in line with previous reports showing that EZH2 is crucial for maintaining a stem cell phenotype in malignant glioma [9], [10]. Both, the specific *EZH2* knockdown by siRNA and the treatment with 5 µM DZNep led to a strong reduction of the trimethylation of H3K27 in human U87MG glioma cells confirming the inhibition of the EZH2 histone methylation activity (Figure 2G). As opposed to *EZH2* knockdown, however, treatment with DZNep did not result in suppression of invasiveness in Boyden chamber assays (Figure 2H), indicating that the antiinvasive effects of the *EZH2* knockdown are not directly mediated by the methyltransferase activity of the PRC2.

### Transcriptional profiling of EZH2-knockdown in human malignant glioma cells

**Figure 3:**
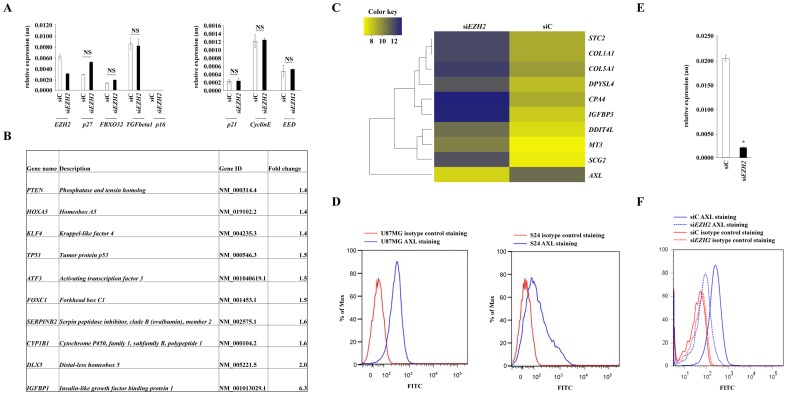
Transcriptional profiling of EZH2-knockdown in human malignant glioma cells. **A** mRNA expression of various known EZH2 target genes 120 h after siRNA mediated *EZH2* knockdown in U87MG glioma cells. **B** Table of known EZH2 target genes, which were regulated as indicated in response to *EZH2* knockdown compared to control in U87MG glioma cells. **C** Heatmap of the ten most regulated genes in response to specific knockdown of EZH2 by siRNA after 120 h in U87MG cells. Upregulated genes are indicated in blue, downregulated genes in yellow. **D** AXL protein expression in the human glioma cell line U87MG (left) and the human GIC S24 (right) by flow cytometry. The cells were stained with an AXL specific antibody: blue or an isotype control: red. **E** *AXL* mRNA expression levels in U87MG human glioma cells incubated for 120 h with scrambled control siRNA (siC) or *EZH2* specific siRNA (si*EZH2*). **F** Analysis of AXL protein expression in U87MG glioma cells treated with siC (solid line) or si*EZH2* (dashed line) for 120 h stained with a AXL specific antibody: blue, isotype control: red. Asterisk indicates * (p<0.05). Error bars indicate s.e.m. NS: not significant.

To analyze the molecular mechanisms that mediate the antiinvasive effects of *EZH2* knockdown we transcriptionally profiled genes that have previously been identified as genes suppressed by EZH2-mediated histone methylation. Surprisingly, *p27*, *p21*, *p16*, *FBXO32*, *EED*, *Cyclin E* and *TGF-β-1* were not induced in *EZH2* knockdown cells (Figure 3A) further suggesting that EZH2 influences the motile phenotype of glioma cells independent of histone methylation. We thus conducted gene microarray analyses of U87MG glioma cells after *EZH2* knockdown. In total 1496 genes were downregulated and 1695 genes were upregulated 120 h after *EZH2* knockdown in U87MG cells. Of the induced genes were genes known to be methylated and silenced by EZH2 such as *TP53*, *ATF3*, *FOXC1*, *SERPINB2*, *CYP1B1*, *DLX5* and *IGFBP1* (Figure 3B). The most downregulated gene was the receptor tyrosine kinase AXL, which has previously not been implicated as an EZH2 target gene (Figure 3C). AXL baseline expression was analyzed in the glioma cell line U87MG and the GIC S24 by FACS (Figure 3D). Furthermore qRT-PCR and FACS analyses confirmed the suppression of *AXL* mRNA and protein expression in U87MG cells after *EZH2* knockdown (Figure 3E, F).

### Methylation-independent regulation of AXL by EZH2

**Figure 4:**
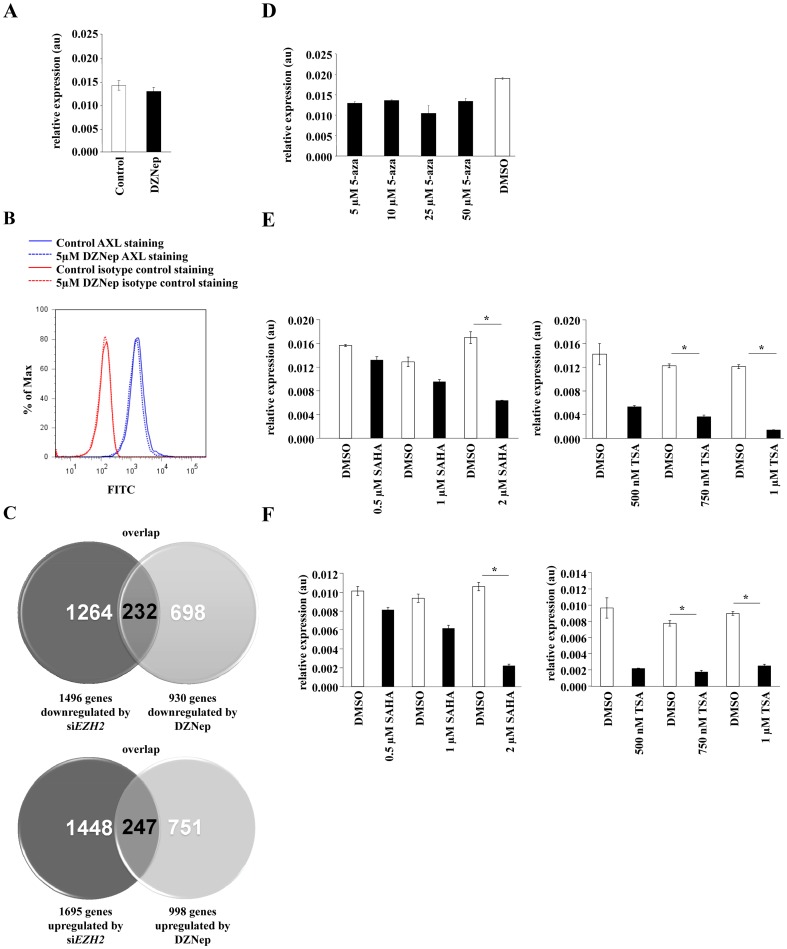
Methylation independent regulation of AXL. **A** *AXL* mRNA expression of U87MG human glioma cells treated with 5 µM DZNep for 120 h in comparison to control. **B** Analysis of AXL protein expression in U87MG glioma cells untreated (solid line) or treated with 5 µM DZNep for 120 h (dashed line) stained with an AXL specific antibody (blue) or an isotype control antibody (red). **C** Comparison of genes with decreased (upper Venn-diagram) or increased (lower Venn diagram) expression after 120 h of *EZH2* knockdown (purple) or DZNep treatment (blue) of U87MG glioma cells. Cutoff: 1.5 fold change. **D** *AXL* mRNA expression of U87MG human malignant glioma cells after 96 h treatment with the DNA methylation inhibitor 5-aza-2′-deoxycytidine (5-aza) (black bars) or DMSO (white bar). **E** *EZH2* and *AXL* mRNA expression of U87MG glioma cells after the stimulation with indicated concentrations of the histone deacetylase inhibitors suberoylanilide hydroxamic acid (SAHA) or trichostatin A (TSA) (black bars) or DMSO (white bars) for 24 h. Asterisk indicates * (p<0.05). Error bars indicate s.e.m.

Next, we analyzed the effect of DZNep on *AXL* mRNA and protein expression by qRT-PCR and FACS. Interestingly no influence of DZNep on the AXL expression was detected, suggesting that the EZH2 mediated regulation of AXL might be methylation-independent (Figure 4A, B). To evaluate our hypothesis that EZH2 also acts through methylation-independent mechanisms we performed a gene microarray assay of U87MG glioma cells treated with DZNep. 930 genes were downregulated and 998 genes were upregulated after DZNep treatment for 120 h. Next the results were compared with the results of the *EZH2* knockdown microarray and we found less than 20% overlap between the two groups (Figure 4C) -a further indication that EZH2 is able to act independent of its histone methylation properties. Since EZH2 is also capable of binding DNA methyltransferases and therefore influencing the DNA methylation, we treated U87MG cells with the DNA methylation inhibitor 5-aza-2′-deoxycytidine (5-aza). 5-aza failed to modulate *AXL* expression in glioma cells (Figure 4D) further supporting the notion that EZH2 controls AXL expression in a histone- and DNA methylation-independent fashion. As polycomb proteins have been shown to interact with histone deacetylase (HDAC) to regulate gene transcription [29]–[31], we analyzed, whether HDAC inhibition altered *AXL* mRNA expression. Indeed, treatment with the histone deacetylase inhibitors trichostatin A (TSA) or suberoxylanilide hydroxamic acid (SAHA) led to a suppression of *AXL* mRNA expression (Figure 4E). Interestingly, HDAC inhibition was equally capable of suppressing expression of *EZH2* itself (Figure 4F).

In addition, it has been reported that a crosstalk between PRC2 and histone 3 lysine 4 (H3K4) demethylases leads to a regulation of histone methylation and thereby to a fine-tuning in the transcriptional control of different target genes [32]. In contrast to H3K27 methylation, methylation of H3K4 is associated with transcriptional activation [33]. To rule out that the depletion of EZH2 leads to an upregulation of H3K4 demethylases resulting in the increase of *AXL*, the effect of the specific *EZH2* knockdown and DZNep treatment on the expression levels of the JARID1 family was analyzed by qPCR. A significant change in mRNA expression of the investigated H3K4 demethlyases could not be detected (Figure S2), suggesting that H3K4 demethylases are not involved in the EZH2 mediated AXL regulation.

### Expression of AXL in human malignant glioma and antiinvasive effect of AXL in human malignant glioma cells

**Figure 5:**
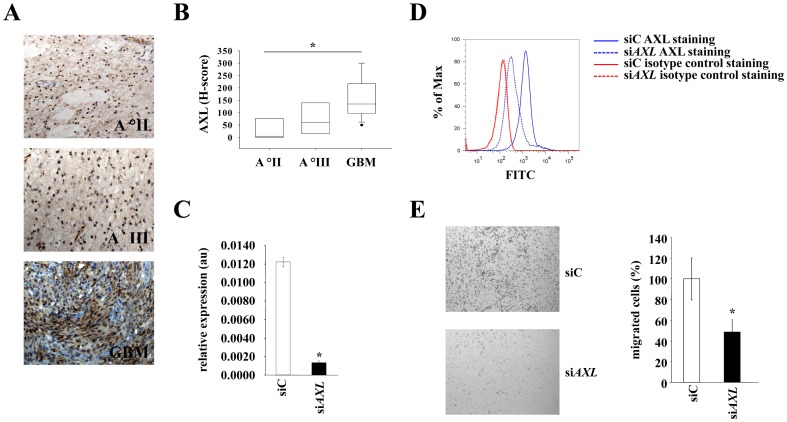
Expression and functional relevance of AXL in human malignant glioma. **A** AXL expression (brown) in sections of human malignant brain tumors with different grades: astrocytoma grade II (top), astrocytoma grade III (middle), glioblastoma (bottom). Magnification 200x. **B** Box-plot of AXL expression in human brain tumors of increasing malignancy (WHO grade II: n = 4, mean = 27.5, SD = 48.56; WHO grade III: n = 5, mean = 74, SD = 65.42; GBM: n = 12, mean = 160.83, SD = 80.96; r^2^ = 0.391, P = 0.002). **C** *AXL* mRNA expression in U87MG glioma cells treated with scrambled control- or *AXL* specific-siRNA for 72 h. **D** Analysis of AXL protein expression in U87MG glioma cells incubated with siC (solid line) or si*AXL* for 120 h (dashed line). AXL specific antibody: blue, isotype control antibody: red. **E** Invasion of U87MG glioma cells with transient *AXL* knockdown (lower panel, black bar) through a matrigel-coated boyden chamber in comparison to control (upper panel, white bar). Asterisk indicates * (p<0.05). Error bars indicate s.e.m.

To examine the pathophysiological relevance of AXL in human malignant glioma we evaluated AXL expression in brain tumor tissue by immunohistochemistry. AXL expression was found in human gliomas with EZH2 expression and is also positively correlated with the grade of malignancy (Figure 5A, B; r^2^ = 0.391, P = 0.002). To determine whether the antiinvasive effect of *EZH2* knockdown in malignant glioma cells is mediated by AXL we used siRNA to inhibit AXL expression (Figure 5C, D). Knockdown of *AXL* led to a profound suppression of glioma cell invasiveness in Boyden chamber assays (Figure 5E), thus mimicking the phenotype of *EZH2* knockdown cells. Collectively, these data indicate that EZH2 controls glioma cell invasiveness *via* AXL independent of DNA and histone methylation.

## Discussion

AXL is a multifunctional receptor tyrosine kinase implicated in neural and mesenchymal development. AXL has been shown to be involved in tumor invasiveness and metastases in multiple tumors [34], [35], [36], [37], [38] including glioma [39], [40], [41]. The molecular mechanisms by which AXL controls tumor invasiveness remain elusive. In mesothelioma AXL-mediated invasiveness involves the PI3K/AKT/mTOR pathway [36], [42], a pathway that is currently targeted in glioblastoma early drug development [43]. In fact the PI3K/AKT/mTOR pathway may be required for at least some of the tumor-promoting effects of AXL [44]. In addition, AXL may signal through NF-κB and matrix metalloprotease 9 (MMP 9) to drive tumor invasion [45]. AXL is activated in tumor cells by growth arrest specific gene 6 (Gas6), a soluble serum protein, in an autocrine fashion [34], [46]. Consequently blocking the Gas6/AXL signaling pathway represents an attractive therapeutic strategy in cancer [47], [48], [49], [50].

The molecular mechanisms constituting overexpression of AXL in cancer are still elusive. In breast cancer, AXL expression is driven by proteins associated with epithelial-to-mesenchymal-transition (EMT) [46], [51]. Interestingly, EZH2 is required for E-cadherin repression by Snail1 during EMT, providing another potential link between EZH2 and AXL [52]. Conversely *AXL* is negatively regulated on a transcriptional level by miR-34a and miR-199a/b [53]. Using the miRNA prediction programs Target Scan and PicTar we found no *AXL* regulating miRNAs, which are known to be regulated by EZH2 [54], [55] . Our findings indicate that EZH2 activates transcription of *AXL* mRNA in a methylation independent manner. This is supported by the fact that treatment with DZNep did not affect AXL expression in glioma cells. While DZNep has originally been described as specific inhibitor of trimethylation of lysine 27 on histone H3 (H3K27me3) and lysine 20 on histone H4 (H4K20me3) mediated by the PRC2 complex [56], more recent studies indicate that DZNep rather globally suppresses histone methylation [57]. Inhibition of histone trimethylation by DZNep not only depletes EZH2 by inducing its proteasomal degradation but also other proteins of the PRC2 complex such as *suppressor of zeste homolog 12* (SUZ12), *embryonic ectoderm development* (EED) and *DNA (cytosine-5)-methyltransferase 1* (DNMT1) [56], [58], [59]. In line with our results (Figure 4C) comparative studies have indicated that there is a surprisingly small overlap of genes regulated by PRC2 silencing and DZNep treatment [56]. As PRC2 proteins including EZH2 primarily epigenetically silence genes there has not been a comprehensive analysis of genes that are positively regulated by EZH2 in cancer. It is possible that other components of the PRC2 such as SUZ12 or EED silence *AXL* mRNA expression while EZH2 positively regulates AXL. Hence, depletion of other PRC2 components, which differentially induce silencing of target genes such as *AXL* by histone methylation may mask the positive effect of EZH2 on *AXL* transcription. While early studies have indicated that the proinvasive effect of EZH2 requires a functional SET domain [62], there is now increasing evidence that EZH2 is capable of transactivating target genes involved in cellular proliferation such as *c-myc* and *cyclin D1* independent of its SET domain by physically interacting with receptor-mediated pathways in cancer cells [63]. In glioblastoma tissue we observed a strong EZH2 expression in perinecrotic areas (Figure 1C). Importantly, these cells are known to migrate away from hypoxic areas. It may well be possible that EZH2 specifically regulates cell motility through transactivation of target genes such as AXL not only in cancer cells but also in stem cells during development. Further studies will focus on SET domain-independent functions of EZH2 and other components of the PRC2. In line with previous results [8], [61], we found strong EZH2 expression in GBMs whereas the expression was low in astrocytoma grade III and absent in astrocytoma grade II (Figure 1A, B). There was no association of EZH2 expression with the IDH1 mutational status (data not shown). The weak EZH2 expression in low grade gliomas (Figure 1A, B) could possibly be due high presence of miR-101 which is known to repress EZH2. It has been shown that miR-101 is downregulated in GBMs resulting in an overexpression of EZH2 [64]. Compared to EZH2, we found a stronger expression of AXL in astrocytoma grade II and III. This could be explained by the presence of other known regulators besides EZH2 of AXL such as SP1/2 and AP-1 [65], [66]. Additionally it was shown that also other epigenetic regulation mechanisms such as promoter methylation are involved in the regulation of AXL [36], [60]. Previous studies in cancer and stem cells suggest that HDAC inhibition suppresses transcription of polycomb proteins and results in a depletion of EZH2 [8]. While we initially postulated that HDAC are directly involved in mediating modulation of *AXL* transcription by EZH2 in gliomas, our data suggest that inhibition of HDAC suppress *AXL* transcription by transcriptional control of *EZH2*, indicating that EZH2 is under transcriptional control of HDAC also in malignant glioma. Although the exact molecular mechanisms driving *AXL* gene expression through EZH2 remain elusive, the identification of AXL as a novel target of EZH2 adds further evidence to the molecular network influenced by EZH2 to sustain the malignant phenotype of tumors.

## Supporting Information

Figure S1 **Culture of glioma initiating cells.** The human GIC S24 and T269 form neurospheres under the indicated culture conditions.
(TIFF)

Figure S2 **Influence of EZH2 depletion on the expression of H3K4 demethylases.** Expression of *JARID1A-D* mRNA after the specific knockdown of EZH2 by siRNA (right) or after the treatment with 5 µM DZNep (left) for 72 h. Error bars indicate s.e.m. NS: not significant.
(TIF)

Table S1 **QPCR primer sequences.**
(TIF)
